# Does 22q11.2 deletion syndrome contribute to the genetic aetiology of Parkinson’s disease?

**DOI:** 10.1007/s00415-018-9046-x

**Published:** 2018-09-20

**Authors:** W. Fung, K. J. Peall

**Affiliations:** 1 Department of Neurology, Institute of Psychological Medicine and Clinical Neurosciences, Cardiff University, Cardiff, CF14 4XW UK; 2 Division of Psychological Medicine and Clinical Neurosciences, Neuroscience and Mental Health Research Institute, Cardiff University, Cardiff, UK

## Introduction

Parkinson’s disease (PD) is the most common neurodegenerative movement disorder with several disease-causing genes now identified. More recently an association between early-onset Parkinson’s disease (EOPD) and deletions involving the 22q11.2 chromosomal region (22q11.2 deletion syndrome, 22q11.2DS) has been described. This represents one of the most common interstitial deletion syndromes (1 in 4000 live births), and spans a 1.5–3 Mb region. 22q11.2DS is a heterogenous clinical syndrome involving neurodevelopmental, psychiatric, cardiac, immunological, and endocrinological abnormalities, with an autosomal dominant inheritance pattern, although many reported cases represent de novo mutations. The area of interest contains ~ 52 genes, none of which have been previously linked to PD. The papers discussed below provide evidence of a potential association between 22q11.2DS and EOPD, and further seek to delineate the characteristic clinical features of this group.

## Association between early-onset Parkinson disease and 22q11.2 deletion syndrome

### Identification of a novel genetic form of Parkinson disease and its clinical implications

Butcher et al. were the first to build on anecdotal evidence of an increased incidence of PD in those with 22q11.2DS. Their systematic assessment involved: (1) determining the frequency of PD amongst a cohort of individuals with 22q11.2DS, (2) genetic screening for the 22q11.2 deletion in individuals diagnosed with young-onset PD, and (3) post-mortem brain tissue analysis of individuals diagnosed with 22q11.2DS and PD during life to determine whether the histopathological changes mirrored those seen in idiopathic PD.

Initial analysis involved clinical data from a large adult Canadian cohort identified as having a hemizygous 22q11.2 deletion (*n* = 159). Recruitment was biased towards the younger and more severe end of the phenotypic spectrum, with individuals recruited via congenital, cardiac, psychiatric and genetic services, and ranging in age between 18 and 68.6 years. They sought to identify the number of individuals with a confirmed clinical diagnosis of PD, demonstrating a significantly higher rate compared to the general population (standardised morbidity ratio = 69.7; 95% CI 19.0–178.5). Four patients were identified with early onset features (39–48 years), with a typical motor clinical phenotype, treatment response, and time course to that seen in idiopathic PD. There was evidence of lateralised motor signs in 2/4 (50%), and all responded to treatment with dopaminergic therapy. Three of these patients had previously been treated with antipsychotic medication, with this contributing to an estimated delay in diagnosis of up to 10 years. There was no family history of PD in any of the cases, and screening for *LRRK2, PARK2, PARK7, PINK1*, and *SNCA* gene mutations was negative. In the overall cohort only one patient was > 65 years, and therefore, it remains possible that further individuals will develop PD later in life.

Of the original cohort of 159, 18 individuals had died at the time of data analysis, with post-mortem data available for six, three of whom had a clinical diagnosis of PD in life. Neuropathological findings were compared between PD and 22q11.2DS (*n* = 3), 22q11.2DS alone (*n* = 3), and age-and sex-matched control group (*n* = 10). These results demonstrated histopathological changes consistent with that of idiopathic PD, namely loss of midbrain dopaminergic neurons and extensive nigral degeneration, in all three cases of 22q11.2DS-associated PD. Of these, α-synuclein-positive Lewy bodies were also present in two of the three cases (corresponding to Braak stages V and VI). Finally, one 22q11.2DS case was identified after screening 225 patients with early-onset PD using quantitative polymerase chain reaction (qPCR) techniques. However, this patient was subsequently identified to be patient 3 in the 22q11.2DS cohort.

*Comment*. This study was the first to demonstrate the potential link between 22q11.2DS and early-onset PD. Clinical similarities to idiopathic PD included motor phenotype, response to Levodopa treatment and neuropathological findings. However, the 22q11.2DS cohort was biased towards the more severe clinical phenotypic and younger age of the disorder spectrum, potentially not accurately reflecting PD prevalence amongst this group. Additionally, genetic testing of the EOPD revealed only a single previously known case, potentially raising doubts as to the frequency of this genetic aetiology amongst those presenting initially with a PD motor phenotype.

Butcher et al., JAMA Neurol 2013; 70(11): 1359–1366.

### Deletions at 22q11.2 in idiopathic Parkinson’s disease: a combined analysis of genome-wide association data

This study took the opposite approach to that of the first paper, estimating the frequency of 22q11.2DS in a large cohort of individuals with PD. In total, 9,387 PD patients and 13,863 controls were analysed from four independently recruited cohorts: (1) the UK Wellcome Trust Case Control Consortium 2 Parkinson’s Disease (*n* = 1592), (2) Dutch Parkinson’s Disease Genetics Consortium (*n* = 740), (3) US National Institute on Aging (NIA) (*n* = 593), and (4) the International Parkinson’s Disease Genomics Consortium (IPDGC NeuroX) (*n* = 6462). All recruited participants fulfilled UK Brain Bank Criteria for the diagnosis of PD, and were negative when screened for mutations in known PD-causing genes.

A 22q11.2 deletion was identified in eight unrelated individuals, from three of the four cohorts, all spanning > 3 Mb, with none identified in the control sample. Interestingly, located within the proximal region of the deleted region, is the *COMT* gene, implicated in dopamine metabolism. Mean age at onset of parkinsonian symptoms was 18 years younger in those with a 22q11.2 deletion compared to those without (42 and 60 years, respectively) with a significantly higher frequency of 22q11.2 deletions in those with EOPD (five patients ≤ 45 years, *p* < 0.0001) as well as a smaller association in those with late onset motor symptoms (three patients > 45 years, *p* = 0.016). Four had abnormal dopamine transporter imaging (DAT scan), suggesting that the observed clinical parkinsonian features were due to PD, rather than prior neuroleptic medication, although detailed medication usage was absent in the majority. Of the available data, PD patients with 22q11.2DS demonstrated symptomatic improvement to dopaminergic therapy (*n* = 5), although tended towards faster symptomatic deterioration and had an early onset of drug-induced dyskinesias.

*Comment*. This study identified eight patients with 22q11.2DS-associated PD, but was limited by its cross-sectional, retrospective nature, and at times limited clinical data. A larger factor however, was the inconsistent exclusion of known PD gene mutations; no data was reported in the Dutch study, only *PARK2* and *PINK1* mutations were screened for in the UK and US-NIA studies. The lack of major cardiac abnormality or schizophrenia in the PD group, and the absence of deletions seen in the control group potentially indicate recruitment bias, influencing the associations identified.

Mok et al., Lancet Neurol 2016; 15:585–596.

### Typical features of Parkinson disease and diagnostic challenges with microdeletion 22q11.2

This international observational study is the largest to summarise the clinical features and neuroimaging findings of 45 individuals with 22q11.2DS-associated PD. A total of 23 publications involving 35 patients were identified. Clinical information was collected using standardised data forms, either from medical records or direct assessments (26/35, 74.3%), with 10 further unpublished cases from the International Consortium on Brain and Behaviour in 22q11.2DS.

Overall, 22q11.2DS-associated PD was more frequently observed in men (71.1%), mean age at onset of motor symptoms was 39.5 years, with 71% of the patients meeting EOPD criteria. Sixteen patients (84%) had a de novo deletion, with mean age at PD diagnosis being 42 years. Individuals with a history of antipsychotic use had a longer median time to PD diagnosis (5 years) compared to antipsychotic-naive patients (1 year). As seen in idiopathic PD, patients ultimately developed increased gait impairment and bulbar dysfunction, and all patients demonstrated rigidity in the final assessment. New onset of neuropsychiatric symptoms was thought to be related to antiparkinsonian medications in eight patients, however, one cannot exclude PD progression, or as a result of the deletion syndrome. Early-onset dystonia, intellectual disability, schizophrenia and seizures were other features more often seen in 22q11.2DS-associated PD.

The majority of patients demonstrated a positive response to levodopa medication (28/30, 93%) and dopamine agonists (12/14, 86%). Five patients received Deep Brain Stimulation, with symptomatic improvement in four. Seven patients had died at the time of data analysis, with a median age at death of 56 years. Presynaptic dopaminergic imaging information was available for 20/35 patients (44.4%). Eighteen of these were DAT scans, with 17 demonstrating reduced (contralateral or bilateral) striatal binding. The remaining two patients underwent PET and 11C-dihydrotetrabenazine ([11C]DTBZ) scans, one of which demonstrated reduced striatal binding.

*Comment*. Although this study represents an extensive international collaborative effort, and the largest data collection to date, the authors identify a number of potential limitations: potential publication bias given the method of case identification, under-representation of parkinsonian symptoms given the younger age of the cohort, incomplete data sets and risk of mis-interpretation of case note documentation. These highlighted the need for ongoing prospective recruitment of cases on an international basis, with longitudinal data collection, and equal emphasis on non-motor as well as motor information.

Boot et al., Neurology 2018; 90:e2059–2067.

